# Research on Transmission Characteristics of Magnetic Couplers for Underwater Wireless Power Transfer Based on Prior Knowledge Input Neural Network

**DOI:** 10.3390/s26051712

**Published:** 2026-03-08

**Authors:** Jixie Xie, Chong Zhu, Xi Zhang

**Affiliations:** School of Mechanical Engineering, Shanghai Jiao Tong University, Shanghai 200240, China; xiejixie@sjtu.edu.cn (J.X.); braver1980@sjtu.edu.cn (X.Z.)

**Keywords:** underwater wireless power transfer, magnetic coupler, power loss modeling

## Abstract

Underwater wireless power transfer (UWPT) operates under special conditions, where the conductivity of seawater introduces eddy current losses, thereby reducing system efficiency. Meanwhile, the design parameters of magnetic couplers significantly influence their transmission characteristics. This paper proposes a fast and accurate neural network prediction model for mutual inductance and losses of magnetic couplers based on mirror-method prior knowledge within a prior knowledge input (PKI) framework. The proposed model integrates a low-fidelity analytical model with data-driven learning to achieve high prediction accuracy while maintaining computational efficiency. Based on the developed model, the transmission characteristics of unipolar rectangular and bipolar DD magnetic couplers are systematically investigated. The results indicate that the rectangular couplers exhibit higher overall efficiency than the DD couplers, with a more monotonic variation in efficiency under design constraints. Owing to its structural characteristics, the DD couplers present an optimal current-carrying area ratio, which is approximately 0.85 within the parameter range. Experimental validation is conducted at a 1 kW power with outer dimensions of 200 mm × 250 mm. The optimal transfer efficiencies of the rectangular and DD couplers reach 97.33% and 96.19%, respectively. The experimental results show good agreement with both simulations and model predictions, demonstrating the reliability of the proposed method for UWPT magnetic coupler analysis.

## 1. Introduction

With the development of marine science and technology, the demand for ocean exploration, resource prospecting, and hydrological environment monitoring has steadily increased [1,2]. Underwater platforms equipped with sensors, including buoy systems, underwater robots, autonomous underwater vehicles (AUVs), and fixed underwater sensor networks, are required to operate for extended periods in marine environments. To enhance their endurance and operational range, power supply for underwater devices and sensing systems has become a major research focus [3]. At present, charging is primarily realized through cable connections, which suffer from high operational costs, the need for manual intervention, and potential safety hazards such as short circuits and electrical leakage in conductive seawater [4,5,6]. Wireless power transfer (WPT) enables contactless power transmission through magnetic couplers, eliminating physical connectors and thereby improving the safety and convenience of the charging process. This provides a promising solution for underwater power replenishment [7]. In addition, an AUV can serve as a mobile power source, receiving power from a mothership and subsequently delivering wireless power to fixed underwater sensor networks.

As a key component of underwater wireless power transfer (UWPT) systems, magnetic couplers determine the power capacity through their mutual inductance and influence the overall system efficiency through their associated losses [8]. These two factors constitute critical performance indicators that govern the transmission characteristics of magnetic couplers and therefore require accurate modeling. Although the finite element method (FEM) can provide relatively accurate results, it is computationally time-consuming and inefficient for transmission characteristics analysis and optimization studies [9]. In contrast, analytical methods can reveal the relationships between design parameters and transmission characteristics and can be readily integrated with intelligent optimization algorithms. Ferrite cores enhance magnetic coupling capability and contribute to size reduction; however, they also modify the magnetic field distribution, thereby increasing the complexity of analytical modeling [10].

Existing studies on loss modeling of magnetic couplers with ferrite cores predominantly adopt the mirror method [10,11]. As a widely used approach for modeling magnetic couplers with ferrite cores, the mirror method can be applied to mutual inductance modeling and loss analysis. However, it requires correction of the image currents, and this correction process typically depends on extensive simulation data [12].

In addition to the mirror method, series expressions are mainly used for analytical modeling of mutual inductance in magnetic couplers with ferrite cores. For circular and rectangular configurations, Bessel function expression s and double Fourier series expressions are typically employed, respectively [9,13]. However, this approach often relies on simplifying assumptions, which may lead to relatively large calculation errors, and some methods are only applicable to single-sided ferrite-core magnetic couplers [13]. The vector magnetic potential Poisson equation can also be solved using double Fourier transform and inverse transform method combined with magnetostatic boundary conditions; nevertheless, this method is generally limited to magnetic couplers with infinite ferrite cores [14]. On this basis, a subdomain analytical method has been proposed to model finite-core magnetic couplers by solving Poisson and Laplace equations. However, the subdomain method still relies on FEM in the calculation process and is not well suited for transmission characteristic analysis [15,16].

In recent years, data-driven models have increasingly been applied to magnetic coupler design [17,18]. However, conventional artificial neural networks (ANNs) typically require complex network architectures or large training datasets to achieve high prediction accuracy [19,20]. Moreover, most conventional ANN models focus primarily on mutual inductance modeling, whereas loss modeling involves additional variables such as frequency and current, making the problem more challenging. Energy-aware deep reinforcement learning has also been applied to adaptive transmission optimization in underwater sensor networks [21]; however, such approaches focus on network-layer strategies rather than physical-layer modeling. To reduce the high data acquisition cost associated with conventional ANN approaches, prior knowledge input (PKI) methods incorporate low-fidelity models or physical priors as additional inputs to guide the mapping between design parameters and outputs, thereby reducing the dependence on large-scale training data [22,23]. In addition, PKI models offer improved interpretability, reliability, high accuracy, fast convergence, and ease of implementation [24]. Therefore, in this paper, the mirror method is adopted as a low-fidelity prior model and incorporated into a PKI framework for magnetic coupler modeling in UWPT systems. The proposed method enables high-accuracy and fast prediction of mutual inductance and losses, while remaining compatible with transmission characteristic analysis algorithms.

Furthermore, due to the specific operating conditions of UWPT systems, the conductivity of seawater induces eddy current losses that decrease system efficiency [25]. Consequently, the modeling and analysis of eddy current losses have attracted considerable attention. Existing studies mainly rely on FEM and analytical approaches developed for circular air-core coils [26,27]. FEM is computationally time-consuming and lacks flexibility, whereas analytical methods that neglect the influence of ferrite cores are not suitable for transmission characteristic analysis and optimal design of magnetic couplers with magnetic cores.

Beyond modeling research, the optimal operating frequency has become another important research, as the resonant characteristics in seawater differ from those in air. Frequency detuning in UWPT systems can be induced by the seawater [28]. Based on this phenomenon, studies have shown that the optimal operating frequency of UWPT systems in seawater may exceed the nominal resonant frequency [26]. Increasing the operating frequency can enhance the output power of magnetic couplers, reduce their dimensions, and improve transmission efficiency within a certain range. However, excessively high frequencies lead to significant eddy current losses [7]. The literature [29] analyzed factors influencing the optimal resonant frequency and concluded that the contact area between the coils and seawater is a key determinant. Nevertheless, most existing transmission characteristic studies focus primarily on eddy current losses, lacking comprehensive consideration of the overall losses in underwater magnetic couplers.

To address the above issues, this paper first establishes analytical models for the mutual inductance and losses of magnetic couplers without ferrite cores. The mirror method is then applied to model magnetic couplers with ferrite cores, thereby deriving mathematical relationships between design parameters, mutual inductance, and losses, which are subsequently used for neural network input parameter selection. A high-accuracy and fast prediction model based on prior knowledge input (PKI), integrating electromagnetic field theory with data-driven techniques, is then developed. Based on the proposed model, the transmission characteristics of magnetic couplers under different design constraints are systematically investigated. Finally, an experimental prototype is constructed using the design parameters derived from the transmission characteristic analysis, and experimental validation is conducted to verify the reliability of the proposed model.

## 2. Underwater Wireless Power Transfer System

**Figure 1:**
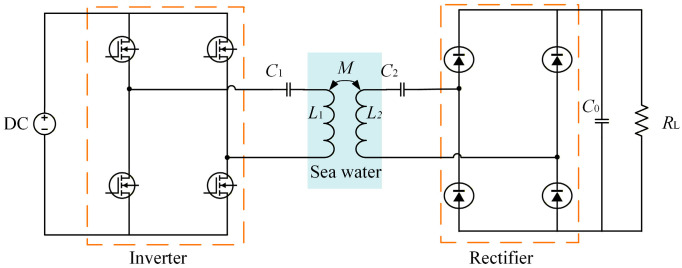
Schematic diagram of UWPT system.

A schematic diagram of a typical underwater wireless power transfer system is shown in Figure 1. Owing to its ability to provide a constant-current output and the relatively small number of required components, the series–series (S–S) compensation topology introduces fewer experimental loss-related error terms, which facilitates a clearer separation of losses in different system components. In addition, the parameter design of the S–S compensation topology is independent of variations in mutual inductance and load conditions. Therefore, the S–S compensation topology is adopted in this study.

When the parasitic resistances of the coils are neglected, the circuit parameters of the S–S compensation topology can be derived according to Kirchhoff’s laws as follows:

(1) $$\left\{\begin{array}{l} I_{1}=\frac{U_{2}}{\omega_{0}M}\\ I_{2}=\frac{U_{1}}{\omega_{0}M} \end{array}\right.$$

where *I*_1_ and *I*_2_ are the root-mean-square (RMS) values of the current of the primary coil and secondary coil respectively, *U*_1_ and *U*_2_ are the ac voltages of the inverter and rectifier respectively, *ω*_0_ is the resonant angular frequency, and *M* is the mutual inductance of the magnetic coupler. The resonant capacitance configuration can be calculated by

(2) $$\left\{\begin{array}{c} C_{1}=\frac{1}{{\omega_{0}}^{2}L_{1}}\\ C_{2}=\frac{1}{{\omega_{0}}^{2}L_{2}} \end{array}\right.$$

where *C*_1_ and *C*_2_ are the compensation capacitors of the primary side and secondary side respectively, *L*_1_ and *L*_2_ are the self-inductances of the primary coil and secondary coil respectively. The output power of the magnetic coupler can be expressed as follows:

(3) $$P_{\mathrm{out}}{=\omega }_{0}{\mathrm{MI}}_{1}I_{2}$$

It can be seen from Equation (3) that accurate modeling of the inductance of magnetic couplers is essential in the design and optimization process. The DC-DC efficiency of an underwater wireless power transfer system is closely related to the losses in both the circuit components and the magnetic couplers. The losses in the circuit section mainly consist of the losses in the inverter, rectifier, and resonant compensation capacitors. Under properly tuned resonant operation achieving zero-voltage switching (ZVS), switching losses are significantly reduced and are therefore approximated as conduction losses in this study. This assumption is commonly adopted in high-efficiency WPT systems [30,31]. The losses of the resonant capacitors can be calculated as following:

(4) $$P_{C}=\frac{\tan\delta \left(f_{0}\right)I^{2}}{\omega_{0}C}$$

where tan *δ*(*f*_0_) denotes the tangent of the capacitor loss angle, which can be obtained from the frequency-dependent curves provided in the manufacturer’s datasheet, *I* is the root-mean-square (RMS) current flowing through the capacitor, and the capacitance *C* is determined by the resonant compensation capacitance calculated using Equation (2). By subtracting the circuit losses from the total DC–DC losses, the losses of the magnetic couplers can be obtained. Accordingly, the transmission efficiency of the magnetic couplers can be expressed as follows:

(5) $$\eta =\frac{P_{\mathrm{out}}}{P_{\mathrm{out}}{+P}_{\mathrm{loss}}}\times 100\%$$

where *P*_out_ is calculated by Equation (3), and *P*_loss_ denotes the loss of the magnetic couplers, which will be detailed in Section 3.

## 3. Inductance Calculation Model of Magnetic Couplers

### 3.1. Inductance Calculation Model of Magnetic Coupler with Air Core

For a rectangular magnetic coupler, it can be simplified into a set of concentric rectangular turns. Based on the principle of electromagnetic superposition, the self-inductance of a multi-turn rectangular coil can be calculated by summing the self-inductance of each single-turn rectangular coil and the mutual inductance between different single-turn coils.

The self-inductance of a single-turn rectangular coil can be calculated by [14]

(6) $$\begin{array}{l} L_{R1}=N-G\\ N=\frac{\mu_{0}}{\pi }\left[\mathrm{aln}\frac{2\mathrm{ab}}{a+d}+\mathrm{bln}\frac{2\mathrm{ab}}{b+d}+2\left(d-a-b\right)\right]\\ G=\frac{\mu_{0}l}{2\pi }(\mathrm{lnr}-\frac{\mu }{\mu_{0}}\frac{\xi }{4})\\ d=\sqrt{a^{2}{+b}^{2}} \end{array}$$

where *a* and *b* are the lengths of the two sides of the rectangular coil, *d* is the diagonal of the coil, *l* is the perimeter of the coil, *r* is the radius of the conductor, *μ* and *µ*_0_ is the permeability of the conductor and the vacuum respectively, and *ξ* is a parameter related to the current distribution characteristics within the conductor cross section.

**Figure 2:**
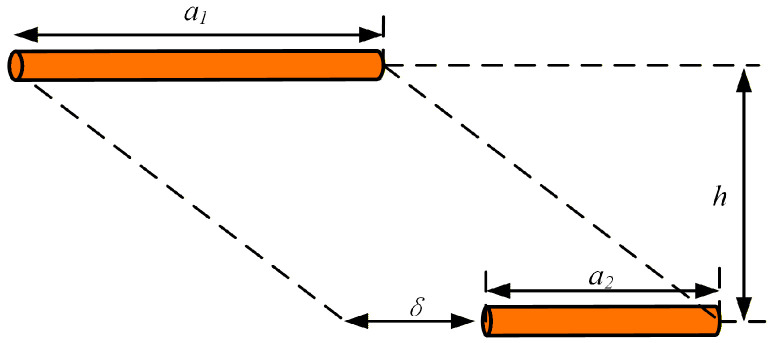
Schematic of Mutual Inductance Calculation for Parallel Conductors.

The mutual inductance between two single-turn rectangular coils can be calculated by summing the mutual inductances of all pairs of parallel conductors within the rectangular coils. As illustrated in Figure 2, two parallel straight conductors with circular cross sections have lengths of *a*_1_ and *a*_2_, respectively, with a length difference of *δ*. The spacing between the two conductors is denoted as *h*. The mutual inductance of the two parallel straight conductors can be calculated using the following formula [32]:

(7) $$\begin{array}{l} M_{\mathrm{PW}}=\begin{array}{c} \frac{\mu_{0}}{4\pi }\left\{\left[\mathrm{Aln}\left(A+\sqrt{A^{2}{+h}^{2}}\right)-\sqrt{A^{2}{+h}^{2}}\right]\right.\\ -\left[\mathrm{Bln}\left(B+\sqrt{B^{2}{+h}^{2}}\right)-\sqrt{B^{2}{+h}^{2}}\right]\\ -\left[\mathrm{Cln}\left(C+\sqrt{C^{2}{+h}^{2}}\right)-\sqrt{C^{2}{+h}^{2}}\right]\\ \left.+\left[\mathrm{Dln}\left(D+\sqrt{D^{2}{+h}^{2}}\right)-\sqrt{D^{2}{+h}^{2}}\right]\right\} \end{array}\\ A=a_{1}+a_{2}+D\;B=a_{1}+D\;C=a_{2}+D\\ D=\left\{\begin{array}{c} \delta -a_{1},\delta \text{>}0\\ -{(a}_{2}+\delta ),\delta \text{<}0 \end{array}\right. \end{array}$$

The mutual inductance of a single-turn rectangular coil can be obtained by summing the mutual inductances between parallel conductors. Accordingly, the self-inductance and mutual inductance of a multi-turn rectangular coil can be calculated by

(8) $$\begin{array}{l} L_{\mathrm{RA}}=\sum_{i=1}^{N}L_{R1}+2\sum_{m=1}^{N}\sum_{n=1}^{N}M_{\mathrm{PW}}\\ M_{\mathrm{RA}}=\sum_{i=1}^{N_{1}}\sum_{j=1}^{N_{2}}\sum M_{\mathrm{PW}} \end{array}$$

where *N*_1_ and *N*_2_ are the turns of primary and secondary coils, respectively. A double D (DD) magnetic coupler consists of two rectangular coils connected in series with opposite winding directions. Therefore, for the DD coils, the self-inductance and mutual inductance can be calculated by

(9) $$\begin{array}{l} L_{\mathrm{DDA}}{=L}_{i1}{+L}_{i2}{+M}_{i1i2}\\ M_{\mathrm{DDA}}{=M}_{p1s1}{+M}_{p1s2}{+M}_{p2s1}{+M}_{p2s2} \end{array}$$

where *i* can be *p* or *s*, denoting the primary or secondary side, respectively. Subscripts 1 and 2 represent the two rectangular coils constituting the DD coils, whose self-inductance and mutual inductance can be calculated using (4–7).

### 3.2. Inductance Calculation Model of Magnetic Coupler with Ferrite Core

Ferrite cores are employed to enhance the transmission efficiency of magnetic couplers and to suppress magnetic flux leakage. However, by guiding stray magnetic flux toward the region between the transmitting and receiving coils, ferrite cores generate strong magnetic flux concentrations around the coils, which makes accurate inductance modeling highly challenging. Therefore, the mirror method is widely used to calculate the self-inductance and mutual inductance of magnetic couplers with ferrite cores.

**Figure 3:**
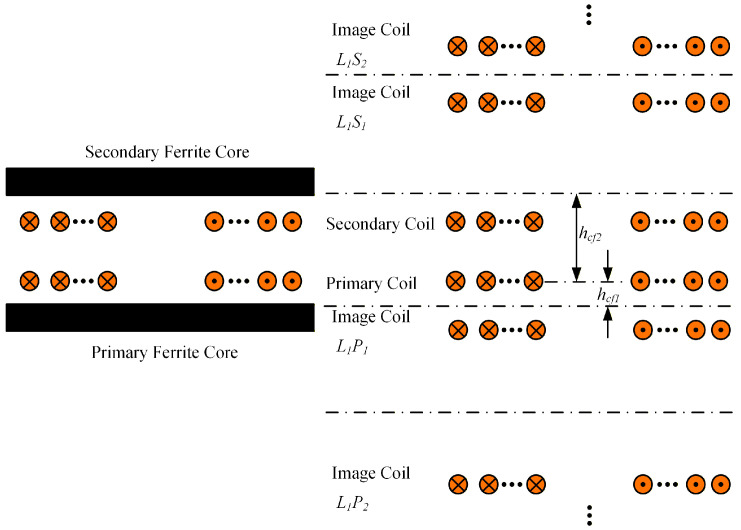
Schematic diagram of the mirror method.

Figure 3 illustrates the principle of the mirror method. The secondary coil remains unchanged, while the upper surface of the primary ferrite core and the lower surface of the secondary ferrite core are selected as the mirror reference planes. First, the primary coil is mirrored with respect to the two reference planes, yielding the image coil *L*_1_*P*_1_ on the primary side and the image coil *L*_1_*S*_1_ on the secondary side. *L*_1_ denotes that the mirror operation is performed on the primary coil, *P* and *S* indicate the primary side and secondary side, respectively, and the subscript 1 represents the first-order image.

Subsequently, the two obtained image coils are further mirrored with respect to the same reference planes, resulting in the second-order image coils *L*_1_*P*_2_ on the primary side and *L*_1_*S*_2_ on the secondary side. This process is repeated iteratively to generate image coils up to the nth order. When the number of image operations exceeds 4 times, the influence of the image coils becomes sufficiently small to be neglected. Therefore, in this study, the image operation is performed twice on both the primary and secondary sides. As the number of image operations increases, the spacing between the image coils can be determined through analytical calculation, as given by

(10) $$\begin{array}{l} h_{L_{1}P_{i}}=\mathrm{ceil}(\frac{i}{2})\times (-2h_{\mathrm{cf}1})-\mathrm{ceil}(\frac{i-1}{2})\times 2h_{\mathrm{cf}2}\\ h_{L_{1}S_{i}}=\mathrm{ceil}(\frac{i}{2})\times (2h_{\mathrm{cf}2})+\mathrm{ceil}(\frac{i-1}{2})\times 2h_{\mathrm{cf}1} \end{array}$$

Under ideal conditions, the mirror method requires the ferrite core to have infinite dimensions. However, this assumption cannot be satisfied in practical applications, as the finite thickness and finite lateral dimensions of ferrite plates inevitably introduce calculation errors. Therefore, a fitting approach that scales the image currents relative to the original currents is commonly employed to reduce these errors. The corresponding fitting coefficient can be given by [10]

(11) $$\left\{\begin{array}{l} F_{1}=\frac{I_{m}}{I_{o}}=\frac{\mu_{r}-1}{\mu_{r}+1}\\ F_{i}=-{F_{1}}^{2i-3}\left(1-{F_{1}}^{2}\right),i\geq 2 \end{array}\right.$$

where *I*_m_ and *I*_o_ are the image current and the original current, respectively, *µ*_r_ is the relative permeability of the ferrite core, and *i* is the number of image operations. Then, the self-inductance and mutual inductance of the magnetic couplers with ferrite cores can be calculated by

(12) $$\left\{\begin{array}{l} {L=L}_{A}{+F}_{1}\cdot M_{L_{1}L_{1}P_{1}}{{+F}_{1}}^{2}\cdot M_{L_{1}L_{1}S_{1}}{{+F}_{1}}^{2}\cdot F_{2}\cdot M_{L_{1}L_{1}P_{2}}{{+F}_{1}}^{2}\cdot {F^{2}}_{2}\cdot M_{L_{1}L_{1}S_{2}}\\ {M=M}_{A}{+F}_{1}\cdot M_{L_{2}L_{1}P_{1}}{{+F}_{1}}^{2}\cdot M_{L_{2}L_{1}S_{1}}{{+F}_{1}}^{2}\cdot F_{2}\cdot M_{L_{2}L_{1}P_{2}}{{+F}_{1}}^{2}\cdot {F^{2}}_{2}\cdot M_{L_{2}L_{1}S_{2}} \end{array}\right.$$

where *L*_A_ is the self-inductance of the magnetic coupler with air core, *M*_A_ is the mutual inductance of the magnetic coupler with air core. The self-inductance and mutual inductance of air-core magnetic couplers, which may adopt either rectangular or DD magnetic couplers, can be calculated using Equations (8) and (9), respectively. $M_{L_{1\;\mathrm{or}\;2}L_{1}{\left(P/S\right)}_{1\;\mathrm{or}\;2}}$ donates the mutual inductance between the primary (*L*_1_) or secondary coil (*L*_2_) and the corresponding image coils. The subscripts of the image coils have been defined in the preceding text.

## 4. Loss Calculation of Magnetic Couplers

### 4.1. Core Loss

In underwater wireless power transfer (UWPT) systems, to achieve high magnetic flux density and compact size, the transmitter and receiver of the magnetic couplers are typically equipped with magnetic cores made of soft magnetic materials such as ferrites. These cores shorten the magnetic path and suppress magnetic flux leakage. The presence of magnetic cores significantly enhances the coupling coefficient; however, it also introduces core loss, which becomes a critical factor affecting overall system efficiency.

Core loss mainly consists of three components: hysteresis loss, eddy current loss, and residual loss. Since the alternating magnetic field in wireless power transfer systems can be reasonably approximated as sinusoidal excitation, the Steinmetz equation is adopted in this study to estimate the magnetic core loss. The Steinmetz equation is obtained by fitting experimental loss curves of magnetic materials over specific ranges of frequency and magnetic flux density, in which the above three loss components are collectively embedded into empirical coefficients and exponents. It is therefore used to calculate the loss of magnetic core materials:

(13) $$P_{\mathrm{core}}=V\cdot \kappa \cdot f_{0}^{\alpha }\cdot {\hat{B}}^{\beta }$$

where *V* is the volume of the ferrite core, $\hat{B}$ is the maximum value of the magnetic flux density in the core, and *κ*, *α*, *β* are the Steinmetz coefficients of the ferrite core. The Steinmetz coefficients depend on the selected ferrite material and its operating temperature. In particular, the coercive force of the ferrite is closely related to the hysteresis component of core loss, as a higher coercivity results in a wider *B*–*H* loop and increased energy dissipation per cycle. The core-loss–frequency characteristic curve at the corresponding operating temperature can be obtained from the manufacturer’s datasheet, based on which the Steinmetz coefficients can be identified by curve fitting. The ferrite core used in this paper is PC40 from TDK Corporation (Tokyo, Japan).

### 4.2. Copper Loss

According to Faraday’s Law of Electromagnetic Induction, when a conductor is subjected to a high-frequency alternating magnetic field, circulating eddy currents are induced within the conductor, generating heat and resulting in eddy current loss. In wireless power transfer systems, Litz wire is commonly used to wind the transmitting and receiving coils in order to reduce coil losses under high-frequency alternating magnetic fields. The winding resistance of Litz wire consists of a DC component and an AC component [33,34]. The DC resistance can be calculated by

(14) $$R_{\mathrm{DC}}=\frac{l}{{\pi n}_{s}r_{s}^{2}\sigma }$$

where *l* is the length of the Litz wire used in the coil, *n*_s_ is the number of strands in the Litz wire, *r*_s_ is the radius of a single strand, and *σ* denotes the electrical conductivity of the conductor.

The AC resistance consists of the resistive components corresponding to eddy currents induced by the skin effect and the proximity effect within the conductor. When the strand diameter of the Litz wire is appropriately selected according to the skin depth, the losses caused by the skin effect can be neglected [35]. Therefore, the AC resistance can be equivalently represented by the proximity-effect resistance and can be calculated using the following expression [11]:

(15) $$P_{\mathrm{AC}}=\sum_{i=1}^{N}\frac{{\pi \sigma \ln}_{s}{d_{s}}^{4}{\omega_{0}}^{2}B^{2}}{128}$$

where *d*_s_ is the diameter of a single strand, and *B* is the flux density across the turn. Therefore, the copper loss of the coil can be demonstrated as

(16) $$P_{\mathrm{copper}}{=P}_{\mathrm{DC}}{+P}_{\mathrm{AC}}{=I}^{2}R_{\mathrm{DC}}{+P}_{\mathrm{AC}}$$

### 4.3. Eddy Current Loss of Sea Water

Unlike wireless power transfer in air, seawater has a conductivity *σ_sea_* of approximately 4 S/m. At higher operating frequencies, the magnetic couplers in UWPT systems induce eddy current fields in the surrounding seawater, which give rise to eddy current losses and consequently reduce the transmission efficiency of the UWPT system. In a seawater medium, there is no external current source. Therefore, the eddy currents density induced in seawater can be calculated by

(17) $$J_{e}=-\sigma_{\mathrm{sea}}\frac{\partial A}{\partial t}$$

where *A* is the magnetic vector potential. The eddy current loss of sea water, *P*_edc_, can be obtained by integrating the product of the squared eddy current density and the seawater conductivity over the seawater region.

### 4.4. Magnetic Field Calculation

The calculation of both core loss and copper loss requires the magnetic flux density in the corresponding regions. The magnetic flux density generated by a finite-length conductor can be calculated based on the Biot–Savart law, and the magnetic flux density of rectangular or DD coils can be obtained by superposition, which is then used to evaluate the associated losses.

**Figure 4:**
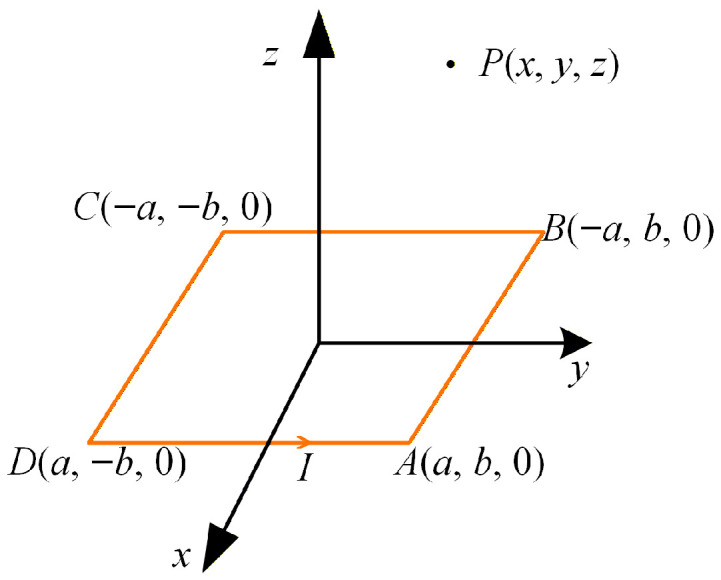
Magnetic vector potential calculation for a single-turn planar coil.

Under the magneto-quasi-static (MQS) assumption, which is valid at the kHz operating frequency of this UWPT system, the magnetic vector potential can be calculated using the Biot–Savart law. For the calculation of eddy current loss, the magnetic vector potential is required and is expressed as

(18) $$A\left(r\right)=\frac{\mu_{0}I}{4\pi }\int \frac{dl}{\left|r-r^{\prime }\right|}$$

where ***r*** denotes the position vector of the observation point, and $r^{\prime }$ denotes the position vector of the source point on the current-carrying conductor. For the planar rectangular coil shown in Figure 4, the magnetic vector potential at the observation point *P* can be calculated by

(19) $$\begin{array}{l} A_{x}\left(x,y,z\right)=\frac{\mu_{0}I}{4\pi }\left[\ln\left(\frac{{a-x+R}_{-+}}{{-a-x+R}_{++}}\right)-\ln\left(\frac{{a+x+R}_{+-}}{{x-a+R}_{--}}\right)\right]\\ A_{y}\left(x,y,z\right)=\frac{\mu_{0}I}{4\pi }\left[\ln\left(\frac{{b-y+R}_{--}}{{-b-y+R}_{-+}}\right)-\ln\left(\frac{{b+y+R}_{++}}{{y-b+R}_{+-}}\right)\right]\\ R_{\text{±±}}=\sqrt{{\left(a\pm x\right)}^{2}+{\left(b\pm y\right)}^{2}{+z}^{2}} \end{array}$$

By applying the superposition principle, the magnetic vector potential distribution of multi-turn rectangular and DD coils in the seawater region can be obtained, and the corresponding eddy current loss can then be evaluated.

For magnetic couplers with ferrite cores, the corresponding magnetic flux density ***B*** and magnetic vector potential ***A*** are obtained by fitting the mirror-method-based results described in Section 3.2, and the associated losses of the ferrite-core magnetic couplers are then evaluated accordingly.

## 5. Prior-Knowledge-Based Mutual Inductance and Loss Model

Although the calculation models presented in Section 2 and Section 3 can approximately evaluate the mutual inductance and losses of magnetic couplers with finite-dimension ferrite cores, the resulting errors remain relatively large. These results will be further discussed with those obtained from the PKI model. Since the value of mutual inductance directly affects the output power of magnetic couplers, a more accurate calculation model is required. Although [11] investigated the influence of core dimensions on the calculation results, no corresponding correction factors were derived, and the calculation error remained close to 15%. The literature [36] proposed correction factors; however, these are only applicable when the coil dimensions are less than 90% of the core dimensions, and are therefore not suitable for the design of compact UWPT magnetic couplers.

**Figure 5:**
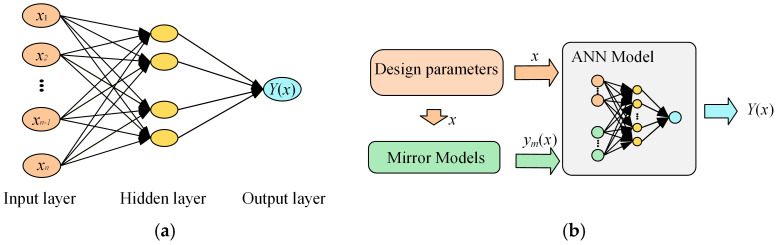
Illustration of neural network models: (**a**) Conventional ANN; (**b**) Prior-knowledge-informed neural network.

To address these limitations, this paper proposes a prior-knowledge-based ANN model for the mutual inductance and losses of magnetic couplers, as illustrated in Figure 5b, which combines mirror models with a backpropagation (BP) neural network. Unlike conventional ANN models as shown in Figure 5a, in addition to using the design parameters of the magnetic couplers related to mutual inductance and loss calculation, denoted by *x*, as inputs, the outputs of the mirror models from the previous two sections, *y*ₘ(*x*), are also incorporated as inputs to the neural network. Specifically, the mirror-model outputs *y*ₘ(*x*) are concatenated with the original design parameters *x* to form an augmented input vector *X*_PKI_ = [*x*, *y*ₘ(*x*)]. The augmented inputs are fed into a fully connected feedforward neural network to predict the high-fidelity target *Y*(*x*). In this manner, the network learns to correct the low-fidelity mirror-model approximation toward the high-fidelity FEM results. The loss function can be expressed as follows:

(20) $$\mathrm{Loss}=\frac{1}{n}\sum_{i=1}^{n}{\left(Y(x\right)-{y(x,y}_{m}\left(x))\right)}^{2}$$

**Figure 6:**
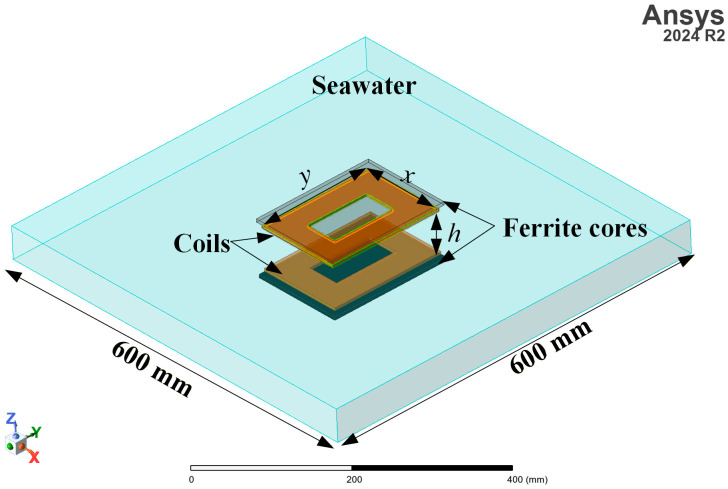
Finite element simulation model of magnetic coupler.

The data used for neural network training are generated from the three-dimensional finite element model (FEM) in ANSYS Maxwell 2024 R2 (Eddy Current solver), as shown in Figure 6. Table 1 lists the design parameters used for training and their corresponding ranges. The parameter ranges listed in Table 1 fully define the sampling space for dataset generation. The upper limit of the RMS current is determined by the selected wire diameter and is set to 20 A, corresponding to a Litz wire with a diameter of 4 mm (0.1 mm × 900 strands). Identical geometric and material parameters are adopted for the primary and secondary magnetic couplers to reduce the dimensionality of the design space and facilitate systematic transmission characteristic analysis. This modeling simplification does not restrict the general framework, which can accommodate asymmetric configurations by introducing independent parameter sets into the input space.

As illustrated in Figure 6, *x* and *y* denote the width and length of the outer dimensions of the magnetic couplers, respectively; *h* is the transfer distance; *I* is the RMS current of the magnetic couplers; *N* is the number of turns; *t*_f_ represents the additional length of the ferrite core beyond the transmitting coil; and *f*_0_ is the operating frequency. The coils are tightly wound, therefore the turn-to-turn spacing is fixed at 1 mm. Once the outer dimensions, number of turns, and turn spacing are specified, the geometric dimensions of the magnetic couplers can be determined. In the FEM, the bottom thickness of the water tank and the thickness of the acrylic supporting plate above the seawater surface were both set to 3 mm to maintain consistency with the experimental configuration.

Latin hypercube sampling (LHS) is employed to generate 500 training samples, which were selected based on the dimensionality of the parameter space and convergence analysis to ensure sufficient coverage while maintaining reasonable computational cost. The corresponding dataset is obtained through FEM simulations. The dataset is divided into training, validation, and test sets with a ratio of 64/16/20. A BP neural network architecture with two hidden layers is adopted, with 32 and 16 neurons in the first and second hidden layers, respectively, to enhance the model’s capability in capturing complex nonlinear relationships. The hyperbolic tangent sigmoid function (tansig) is selected as the activation function for the hidden layers, and the Levenberg–Marquardt (LM) algorithm is used for network training.

**Figure 7:**
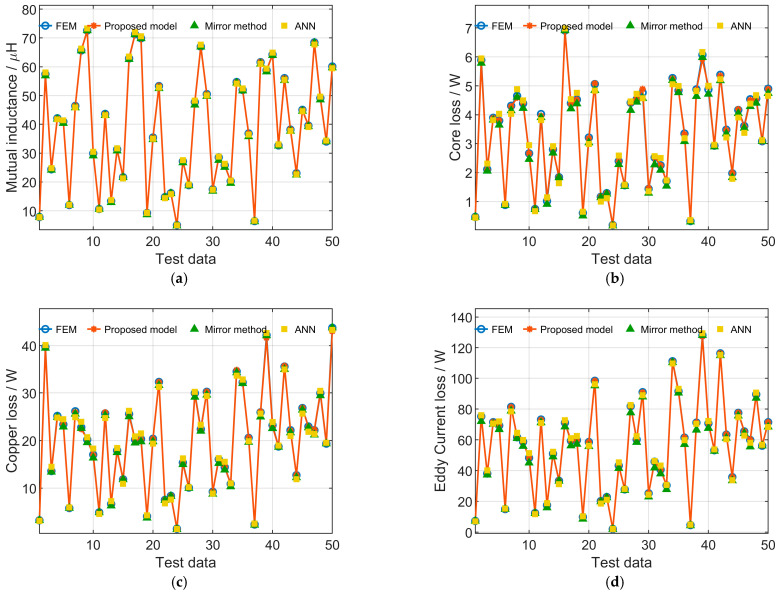
Comparison of test-set results for rectangular magnetic couplers obtained by FEM, the proposed model, the mirror method, and ANN: (**a**) Mutual inductance; (**b**) Core loss; (**c**) Copper loss; (**d**) Eddy current loss.

**Figure 8:**
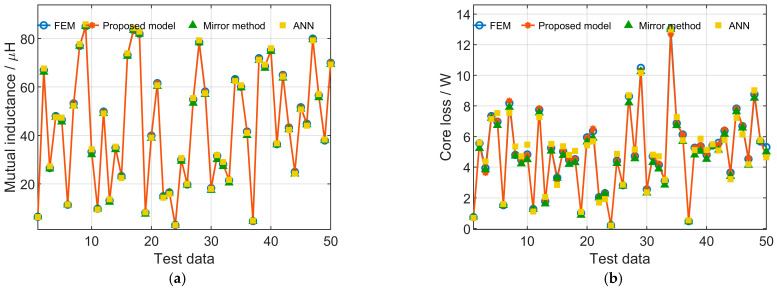

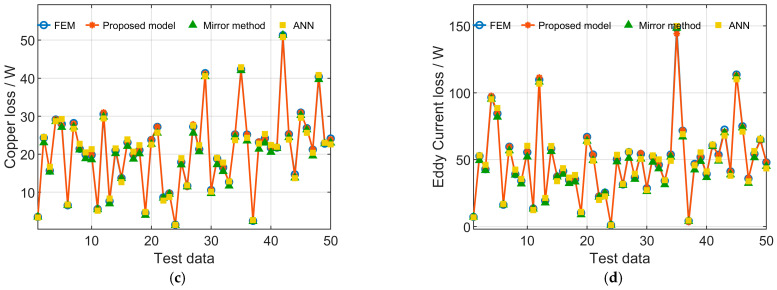
Comparison of test-set results for DD magnetic couplers obtained by FEM, the proposed model, the mirror method, and ANN: (**a**) Mutual inductance; (**b**) Core loss; (**c**) Copper loss; (**d**) Eddy current loss.

To validate the contribution of incorporating prior knowledge, an ablation comparison is conducted under the same training budget, identical data splits, and network architecture, including (i) the analytical mirror model, (ii) the ANN model, and (iii) the proposed PKI-NN model. Figure 7 and Figure 8 present the test-set comparisons for rectangular and DD magnetic couplers, respectively. The results clearly demonstrate that incorporating mirror-model outputs as prior knowledge significantly improves prediction accuracy compared with both the mirror model and the ANN model under identical training conditions, confirming the effectiveness of the PKI mechanism in reducing approximation error and enhancing learning efficiency.

To further evaluate the robustness of the learning-based models, the training process was repeated 10 times with different random data splits. Table 2 summarizes the mean and maximum errors of different models for both rectangular and DD magnetic couplers. The mean errors reported in Table 2 for the ANN and the proposed models are obtained by averaging the results of these repeated experiments, indicating stable training performance. Both the mean and maximum errors of the proposed PKI model are consistently smaller than those of the mirror method and the ANN model, demonstrating improved overall accuracy and better worst-case prediction performance. The relatively larger percentage errors mainly occur in samples with small simulation values, for which the absolute errors are actually small and have a negligible impact on subsequent transmission characteristic analysis. Therefore, the proposed model demonstrates high accuracy and is well suited for the analysis of transmission characteristics of magnetic couplers. Moreover, compared with FEM simulations, which require computation times on the order of minutes for a single case, the trained model achieves a single-case computation time of less than the millisecond level, making it more flexible and efficient for large-scale transmission characteristic analysis.

Table 3 compares the proposed method with existing magnetic coupler modeling approaches based on the mirror method or conventional ANN models. In [10], the mirror method was employed to model the mutual inductance of circular and rectangular magnetic couplers with improved accuracy compared to [32]. However, the computational time per single case is relatively long, which limits its applications. Literature [19] adopted a purely data-driven ANN to model the mutual inductance of circular magnetic couplers. Although the trained model achieves relatively high prediction accuracy and fast inference time, the use of five design parameters as inputs requires a large amount of training data. In contrast, the proposed method requires only 500 training samples to achieve accurate modeling of both mutual inductance and losses of magnetic couplers. Literature [32] applied the mirror method to model the mutual inductance and copper loss of DD couplers. However, compared with the proposed approach, its calculation errors are larger, which reduces its effectiveness for transmission characteristic analysis and magnetic coupler optimization design. It can be observed that works relying solely on the mirror method either suffer from relatively low computational accuracy or require excessive computation time. On the other hand, ANN-based approaches can achieve high prediction accuracy and very short inference time, but they generally depend on a large volume of training data. The method proposed in this paper integrates the advantages of both approaches, achieving high modeling accuracy while requiring only a relatively small training dataset.

## 6. Transmission Characteristics Analysis of Magnetic Couplers

For the transmission characteristic analysis, the parameter dimensionality is reduced by fixing the outer dimension in the *y*-direction to be 50 mm larger than that in the *x*-direction, and setting the transfer distance to 50 mm. The design requirement of the magnetic couplers, i.e., the output power *P*_out_, is categorized into three power levels: 500 W, 1000 W, and 1500 W. The design constraint of the magnetic couplers, i.e., the outer dimension in the *x*-direction, is categorized into three dimension-levels: 150 mm, 200 mm, and 250 mm. The influence of magnetic coupler design parameters on the transmission characteristics is investigated under different combinations of power and size levels.

### 6.1. Transmission Characteristics Analysis Under the Same Power Level and Outer Dimension

**Figure 9:**
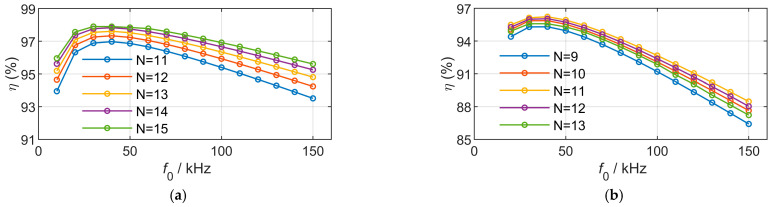
Model results of frequency variation under the same power and outer sizes. (**a**) Rectangular couplers. (**b**) DD couplers.

A reference case with *P*_out_ = 1000 W and an outer x-dimension of 200 mm is selected. By varying the coil turns and operating frequency, the coil current is constrained by the maximum current corresponding to the wire diameter. Meanwhile, the mutual inductance changes with the number of turns, and the operating conditions are adjusted accordingly to maintain an output power of 1000 W. Five sets of design parameters with the highest efficiency are selected, and the efficiency–frequency curves of unipolar and bipolar coils are plotted, as shown in Figure 9.

The results indicate that, for rectangular magnetic couplers, increasing the number of turns leads to a higher mutual inductance. Under the same output power as the mutual inductance increases, it can be found from (3) that the product of the squared current and operating frequency decreases, thereby reducing copper loss and eddy current loss. Consequently, the transmission efficiency increases, and an optimal frequency point exists at approximately 30 kHz.

For DD magnetic couplers, increasing the number of turns can also enhance the coupling capability and thus improve efficiency. However, more turns intensify the magnetic field in the central region, and the effective current-carrying area of DD coils increases more significantly than that of rectangular coils, resulting in higher core loss and eddy current loss. As a result, the transmission efficiency exhibits a nonlinear trend, first increasing and then decreasing with the number of turns. Moreover, the efficiency reduction with increasing frequency is more pronounced than that of rectangular coils.

In addition, for comparable outer dimensions, the mutual inductance of DD magnetic couplers is lower than that of rectangular magnetic couplers. To deliver the same output power, a larger value of the product of operating frequency and squared current is required, which further increases the losses. Therefore, the overall efficiency of DD magnetic couplers is lower than that of rectangular magnetic couplers. The optimal frequency of DD magnetic couplers is slightly higher than that of rectangular magnetic couplers, occurring at approximately 40 kHz.

The operating parameters with the highest efficiency for the two magnetic coupler types are selected for FEM validation. The corresponding parameters are listed in Table 4. The predicted outputs of the proposed model—namely, the mutual inductance and the loss of the magnetic couplers—show excellent agreement with the simulation results, with only negligible percentage errors, demonstrating the reliability of the proposed model.

### 6.2. Transmission Characteristics Analysis Under Different Power Levels

**Figure 10:**
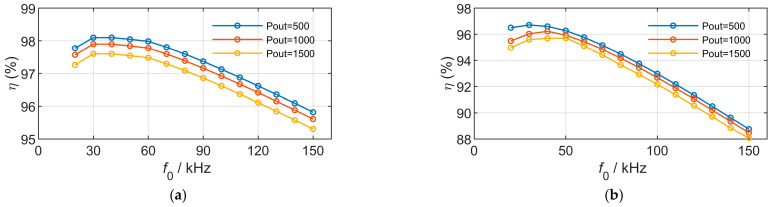
Model results of frequency variation under different output power. (**a**) Rectangular couplers. (**b**) DD couplers.

The curves in Figure 10 are obtained by selecting the design parameters listed in Table 4 and varying the coil output power to investigate the variation in efficiency with operating frequency. When the geometric parameters of the magnetic couplers are fixed (i.e., the mutual inductance is fixed), the product of operating frequency and the squared current is proportional to the output power. As the output power decreases, the current at a given frequency becomes smaller, resulting in lower losses and thus higher efficiency.

At lower frequencies, copper loss accounts for a larger proportion of the total loss, whereas with increasing frequency, the contribution of eddy current loss becomes more significant. For rectangular magnetic couplers, the optimal operating frequency remains unchanged. In contrast, due to the structural characteristics of DD magnetic couplers, their current-carrying area is larger than that of rectangular magnetic couplers, making copper loss and eddy current loss more sensitive to the aforementioned effects.

Under low-power operating conditions, a reduction in operating frequency leads to only a small increase in current. In this case, moderately lowering the frequency and allowing a slight increase in current can reduce the overall loss, resulting in a lower optimal frequency for DD magnetic couplers at low power levels. As the power level increases, changes in frequency lead to more significant variations in current. Appropriately increasing the operating frequency to reduce the current, although it may slightly increase eddy current loss, can yield a much larger reduction in copper loss, thereby improving the overall transmission efficiency. Consequently, the optimal operating frequency of DD magnetic couplers increases at higher power levels.

### 6.3. Transmission Characteristics Analysis Under Different Outer Dimensions

**Figure 11:**
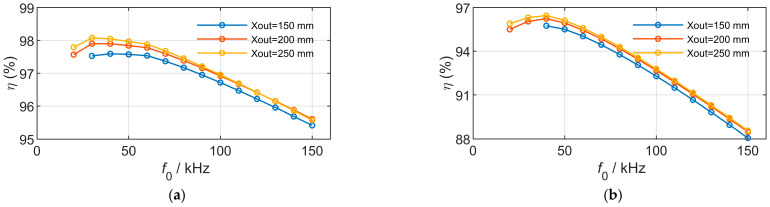
Model results of frequency variation under different outer sizes. (**a**) Rectangular couplers. (**b**) DD couplers.

Similarly, using the design parameters listed in Table 4, the output power is fixed at 1000 W, and the outer dimensions of the magnetic couplers are varied to investigate the efficiency–frequency characteristics, as shown in Figure 11. For the magnetic couplers with an outer dimension of 150 mm, the current corresponding to certain frequency points exceeds the maximum current limit imposed by the wire diameter.

When the transmitted power is kept constant, increasing the outer dimension of the magnetic couplers (i.e., increasing the mutual inductance) reduces the product of operating frequency and the squared current, leading to lower losses and consequently higher efficiency. However, when the operating frequency exceeds a certain range, eddy current loss becomes the dominant component of the total loss. Larger magnetic coupler dimensions also imply a greater contact area between the current-carrying coils and the surrounding seawater, resulting in increased eddy current loss. As a result, the efficiency of larger-dimension magnetic couplers may become lower than that of smaller ones at high frequencies. In addition, as the outer dimension increases, the optimal operating frequency exhibits a decreasing trend.

### 6.4. Transmission Characteristics Analysis with Respect to the Current-Carrying Area

The current-carrying area ratio of the transmitting coil can be defined as

(21) $$Q_{\mathrm{Sc}}=\frac{S_{c}}{S_{\mathrm{out}}}=1-\frac{x_{\mathrm{in}}\cdot y_{\mathrm{in}}}{x_{\mathrm{out}}\cdot y_{\mathrm{out}}}=1-\frac{\left(x_{\mathrm{out}}-2\cdot N\cdot d\right)\cdot \left(y_{\mathrm{out}}-2\cdot N\cdot d\right)}{x_{\mathrm{out}}\cdot y_{\mathrm{out}}}$$

where *x*_in_ and *y*_in_ are the inner dimension of the coils, *x*_out_ and *y*_out_ are the inner dimension of the coils, and *d* is the diameter of the Litz wire. The DD coil consists of two identical rectangular coils; therefore, the ratio can be obtained by calculating that of a single rectangular coil.

For the rectangular coil, within the parameter ranges considered in this study (as listed in Table 1), the optimal current-carrying area ratio always corresponds to the maximum number of turns and remains independent of the power level.

In contrast, for the DD coil, the optimal current-carrying area ratio does not occur at the maximum turn number. It is likewise independent of the power level. As the outer dimension increases, the corresponding optimal turn number increases, while the optimal current-carrying area ratio remains approximately 0.85. Specifically, for outer dimensions of 150 mm, 200 mm, and 250 mm, the optimal numbers of turns are 9, 11, and 14, respectively, corresponding to current-carrying area ratios of 0.854, 0.834, and 0.860.

Therefore, unlike the unipolar rectangular coil, the optimization of the DD coil requires consideration of not only the optimal operating frequency but also the optimal current-carrying area.

## 7. Experimental Verification

### 7.1. Experimental Prototype Setup

**Figure 12:**
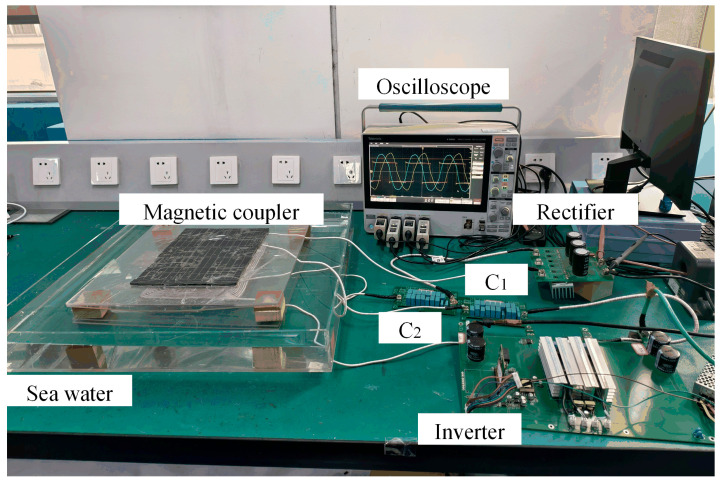
Experimental prototype.

A saltwater solution with a salinity of 3.5% was prepared to emulate the seawater environment. According to the design parameters of the magnetic couplers listed in Table 4, rectangular and DD magnetic couplers were fabricated. A 1 kW experimental prototype was then built, as shown in Figure 12.

### 7.2. Experimental Results

**Figure 13:**
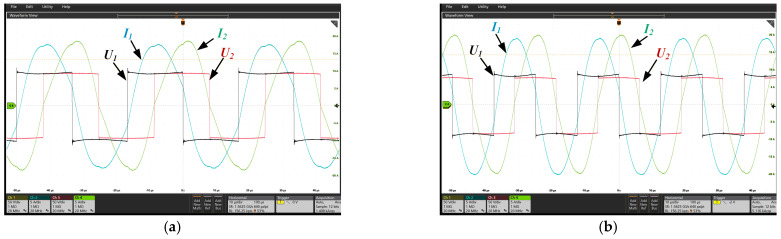
Waveform of the prototype: (**a**) Rectangular coupler; (**b**) DD coupler.

The experimental validation of the rectangular and DD magnetic couplers was conducted in a seawater environment. The inverter output voltage *U*_1_ and current *I*_1_, as well as the rectifier input voltage *U*_2_ and current *I*_2_, are shown in Figure 13a and Figure 13b, respectively. The measured waveforms indicate that the RMS values of the currents during the experiments reach the target RMS currents corresponding to the selected operating conditions, and soft switching is achieved in all cases. Based on these results, the conduction losses of the inverter and rectifier are estimated.

An LCR meter is used to measure the self-inductance and mutual inductance of the magnetic couplers, as well as the equivalent AC resistances of the coils and compensation capacitors. These measurements are employed for configuring the resonant capacitors of the compensation topology and for calculating the losses of the magnetic couplers and compensation capacitors. A power analyzer is then used to measure the DC losses of the magnetic couplers under identical operating conditions in air and in seawater. The difference between these two measurements yields the eddy current loss in the seawater region during the validation experiments. By subtracting all the above losses from the total loss, the core loss is further estimated.

The experimentally measured inductance values of the magnetic couplers, the compensation capacitances, and the loss components, together with the corresponding model predictions, are summarized in Table 5. The discrepancies observed in the measured self-inductance of identical coils are likely attributed to variations in the coil winding process. The experimental results for mutual inductance and transmission efficiency show good agreement with the model predictions, with only small errors, thereby demonstrating the reliability of the proposed model for transmission characteristic analysis.

## 8. Conclusions

Considering the special operating conditions faced by magnetic couplers in UWPT systems, this paper proposes a neural network model for predicting inductance and losses of magnetic couplers based on prior knowledge derived from the mirror method. First, a mirror-method-based analytical model for calculating inductance and losses of magnetic couplers with ferrite cores is established. This analytical model is then incorporated into the neural network as prior knowledge, enabling high-accuracy and rapid prediction of mutual inductance and losses for core-equipped magnetic couplers.

Subsequently, based on the developed model, the transmission characteristics of two commonly used planar magnetic couplers—namely, the unipolar rectangular coil and the bipolar DD coil—are investigated. The results indicate that, under similar operating conditions, the rectangular configuration exhibits slightly higher transmission efficiency than the DD coil, and its efficiency varies more monotonically under different design constraints. For the unipolar rectangular coil, as the current-carrying area increases, the mutual inductance increases, and the transmission efficiency at the optimal frequency improves accordingly. However, as the power level increases, the transmission efficiency of the rectangular coil decreases. Variations in power level also lead to changes in the optimal operating frequency of the DD coil.

Compared with the rectangular coil, the DD coil possesses a larger current-carrying area and, due to its structural characteristics, exhibits a higher magnetic flux density in the central region. Therefore, when the conductor length is comparable to that of the rectangular coil, the DD coil experiences greater overall losses and is more sensitive to variations in design constraints. Moreover, as the current-carrying area varies, the transmission efficiency of the DD coil demonstrates a nonlinear trend—first increasing and then decreasing—indicating the existence of an optimal current-carrying area ratio of around 0.85.

Finally, a prototype is constructed for experimental validation. The experimental results agree well with the model predictions, demonstrating the reliability of the proposed approach. The developed model can be applied to the analysis of transmission characteristics of magnetic couplers, and the results of transmission characteristics analysis of magnetic couplers provide guidance for their optimal design, thereby contributing to the practical application of UWPT systems.

It should be noted that the generalization capability of the proposed model depends on the parameter ranges selected for dataset generation. Although the investigated design space covers typical UWPT operating conditions, extrapolation beyond the trained intervals—particularly under extreme design dimensions, high operating frequencies, or large current amplitudes—may reduce prediction reliability. Future work will extend the model to broader operating domains and improve its robustness under extreme parameter scenarios.

## Figures and Tables

**Table 1:** Range of training data.

Parameter	Value	Parameter	Value
*x*/mm	100–250	*y*/mm	150–300
*h*/mm	10–50	*I*/A	4–20
*N*/turns	5–15	*t_f_*/mm	0–30
*f*_0_/kHz	10–150		

**Table 2:** Error comparison between different methods.

Method	Coupler Structure	Mutual Inductance		Copper Loss		Core Loss		Eddy Current Loss	
		Mean	Max	Mean	Max	Mean	Max	Mean	Max
This work	Rectangular	0.3%	0.6%	1.6%	4%	2.3%	5%	1.5%	3%
	DD	0.5%	1%	1.4%	5%	2.6%	8%	1.8%	4%
Mirror method	Rectangular	3.6%	6%	9.6%	18%	5.6%	12%	6.7%	15%
	DD	5.8%	8%	8.7%	14%	7.2%	15%	5.5%	14%
ANN	Rectangular	1.3%	3%	4.0%	9%	8.5%	17%	5.6%	10%
	DD	2.7%	5%	4.7%	12%	8.2%	21%	4.5%	14%

**Table 3:** Comparison with previous works.

Reference	Coupler Structure	Method	Error	Time of Single Case	Shortcoming to This Work
This work	Rectangular	Mirror-method based PKI-NN	Mean 0.5% (*M*) Mean 2.6% (Loss)	~5 s (Calculation) ~0.01 s (PKINN)	/
	DD				
[10]	Circular	Mirror method	Mean 2.03% (*M*)	4.01 min	Long time consumption
	Rectangular		Mean 4.78% (*M*)	11.48 min	
[19]	Circular	ANN	Mean 3.45% (*M*)	~0.01 s	Large data set (19,874 cases trained)
[32]	DD	Mirror method	<5% (*M*) <10% (Copper loss)	0.59 s (*M*) 6.5 s (Copper loss)	Large error
[11]	Circular	Mirror method	<15% (Copper loss)	/	Large error

**Table 4:** Parameters of selected design.

Parameter	Value (Error/%)		Parameter	Value (Error/%)	
	Rectangular	DD		Rectangular	DD
*x*/mm	200		*y*/mm	250	
*h*/mm	50		*tf*/mm	20	
*N*/turns	15	11	*I*/A	13.2	14.2
*f*_0_/kHz	30	40	*M*/*μ*H	30.44 (−0.12%)	19.65 (+0.05%)
*L*/*μ*H	80.92 (+0.1%)	67.21 (−0.15%)	*P_loss_*/W	21.44 (−1.23%)	39.19 (+0.27%)
*η*/%	97.90	96.23			

**Table 5:** Experimental parameters of underwater wireless power transfer.

Parameter	Rectangular Coupler		DD Coupler	
	Experiment	Model	Experiment	Model
*M*/*μ*H	32.61	30.44	19.88	19.65
*L*/*μ*H	81.79/79.6	80.92	68.74/64.71	67.21
*C*_1_/nF	347.1	/	233.16	/
*C*_2_/nF	320.3	/	230.29	/
*P_loss_*/W	29.38	26.12	40.10	39.20
*η*/%	97.33	97.45	96.19	96.23

## Data Availability

The data can be accessed from this manuscript. Further datasets inquiries are available from the corresponding author upon reasonable request.
